# Chromatin interacting factor OsVIL2 increases biomass and rice grain yield

**DOI:** 10.1111/pbi.12956

**Published:** 2018-06-26

**Authors:** Jungil Yang, Lae‐Hyeon Cho, Jinmi Yoon, Hyeryung Yoon, Antt Htet Wai, Woo‐Jong Hong, Muho Han, Hitoshi Sakakibara, Wanqi Liang, Ki‐Hong Jung, Jong‐Seong Jeon, Hee‐Jong Koh, Dabing Zhang, Gynheung An

**Affiliations:** ^1^ Crop Biotech Institute Kyung Hee University Yongin Korea; ^2^ Graduate School of Biotechnology Kyung Hee University Yongin Korea; ^3^ Plant Productivity Systems Research Group RIKEN Center for Sustainable Resource Science Tsurumi Yokohama Japan; ^4^ State Key Laboratory of Hybrid Rice Shanghai Jiao Tong University–University of Adelaide Joint Centre for Agriculture and Health School of Life Sciences and Biotechnology Shanghai Jiao Tong University Shanghai China; ^5^ Department of Plant Science Research Institute of Agriculture and Life Sciences, and Plant Genomics and Breeding Institute Seoul National University Seoul Korea

**Keywords:** biomass, chromatin interacting factor, grain yield, *OsCKX2*, *OsVIL2*, rice

## Abstract

Grain number is an important agronomic trait. We investigated the roles of chromatin interacting factor *Oryza sativa* VIN3‐LIKE 2 (OsVIL2), which controls plant biomass and yield in rice. Mutations in *OsVIL2* led to shorter plants and fewer grains whereas its overexpression (OX) enhanced biomass production and grain numbers when compared with the wild type. RNA‐sequencing analyses revealed that 1958 genes were up‐regulated and 2096 genes were down‐regulated in the region of active division within the first internodes of OX plants. Chromatin immunoprecipitation analysis showed that, among the downregulated genes, OsVIL2 was directly associated with chromatins in the promoter region of *CYTOKININ OXIDASE/DEHYDROGENASE2* (*OsCKX2*), a gene responsible for cytokinin degradation. Likewise, active cytokinin levels were increased in the OX plants. We conclude that OsVIL2 improves the production of biomass and grain by suppressing *OsCKX2* chromatin.

## Introduction

Increasing grain yield is a major goal in agriculture. In rice (*Oryza sativa*), this outcome is correlated with the numbers of spikelets and branches produced in a panicle (Zhang *et al*., 2013). Panicle branching is controlled by *LAX PANICLE* (*LAX*), encoding a grass‐specific bHLH transcription factor (TF) (Komatsu *et al*., 2003), and *FRIZZY PANICLE* (*FZP*), encoding an ERF TF (Oikawa and Kyozuka, 2009). *DENSE AND ERECT PANICLE1* (*DEP1*) regulates *CYTOKININ OXIDASE/DEHYDROGENASE2* (*OsCKX2*) to enhance meristem activity and increase the number of grains per panicle (Huang *et al*., 2009).

Productivity is also determined by plant architecture. *ABERRANT PANICLE ORGANIZATION1* (*APO1*) expands the size of the inflorescence meristem, leading to increases in culm diameters and spikelet numbers (Ikeda‐Kawakatsu *et al*., 2009; Ookawa *et al*., 2010). *IDEAL PLANT ARCHITECTURE 1* (*IPA1*) and *WEALTHY FARMER'S PANICLE* (*WFP*) encode SQUAMOSA PROMOTER BINDING PROTEIN‐LIKE 14 (OsSPL14), a target of *miRNA156* (Jiao *et al*., 2010; Miura *et al*., 2010). Enhanced expression of *OsSPL14* improves culm thickness and yield (Jiao *et al*., 2010; Miura *et al*., 2010).

Cytokinin plays a fundamental role in regulating the size of reproductive meristems and number of seeds (Jameson and Song, 2016; Kyozuka, 2007). *Grain Number per Panicle1* (*GNP1*) enhances cytokinin levels in the panicle meristems, resulting in more spikelets (Wu *et al*., 2016). *GRAIN NUMBER 1a* (*Gn1a*) is a QTL locus that determine grain yield in rice (Ashikari *et al*., 2005). This gene encodes OsCKX2, which degrades cytokinin. Reduced *OsCKX2* expression increases the level of active cytokinin, resulting in a larger number of tillers and total spikelets per plant (Ashikari *et al*., 2005; Yeh *et al*., 2015). Similarly, homologs of *OsCKX2* in *Hordeum vulgare* and *Triticum aestivum* control panicle size and grain number (Zalewski *et al*., 2010; Zhang *et al*., 2012). Expression of *OsCKX2* is promoted by *LARGER PANICLE* (*LP*) and *DROUGHT AND SALT TOLERANCE* (*DST*). The former encodes a Kelch repeat‐containing F‐box protein found in the endoplasmic reticulum. Mutations in *LP* are associated with taller plants, thicker culms, larger panicles and greater yield (Li *et al*., 2011). Perturbing the zinc finger TF DST reduces *OsCKX2* expression and boosts cytokinin levels, leading to increased plant height and panicle branching, and a consequent improvement in grain numbers (Li *et al*., 2013).

Polycomb repressive complex (PRC) regulates crucial processes in development of animals and plants by silencing target genes via histone modification (Jeong *et al*., 2015; Schuettengruber *et al*., 2007). For example, PRC2 suppresses target loci by enhancing trimethylation of lysine 27 of histone 3 (H3K27) (Cao *et al*., 2002). Arabidopsis has three PRC2‐like complexes: FERTILIZATION INDEPENDENT SEED (FIS), EMBRYONIC FLOWERING (EMF) and VERNALIZATION (VRN), whereas rice has only Arabidopsis EMF2 homologous proteins, OsEMF2a and OsEMF2b (Kohler and Villar, 2008; Luo *et al*., 2009; Schubert *et al*., 2005). The VRN complex interacts with VERNALIZATION INSENSITIVE 3 (VIN3) to form PHD‐PRC2 complex (De Lucia *et al*., 2008; Wood *et al*., 2006), and VIN3 represses *FLOWERING LOCUS C* (*FLC*) during vernalization (Sung and Amasino, 2004). The VIN3 and VIN3‐like proteins (VIL1 through 4) contain conserved motifs of the PHD finger domain, the fibronectin Type III (FNIII) domain and the VIN3 interacting domain (VID) (Greb *et al*., 2007; Sung *et al*., 2006). These proteins function together by binding to each other via the VID domain (Sung *et al*., 2006). Rice has four VIL proteins (OsVIL1 through 4) that have been shown to interact with each other in a yeast system (Fu *et al*., 2007). We have previously reported that OsVIL2 binds to *O. sativa* EMBRYONIC FLOWER 2b (OsEMF2b), a component of rice PRC2 and that PHD‐PRC2 complex induces flowering by repressing *O. sativa LEAFY COTYLEDON 2 AND FUSCA 3‐LIKE 1* (*OsLFL1*) (Yang *et al*., 2013).

Here, we demonstrate that OsVIL2 enhances panicle development and plant yield by reducing active cytokinin levels. We showed that OsVIL2 decreases *OsCKX2* expression by directly interacting to the promoter region of *OsCKX2*.

## Results

### Mutations in *OsVIL2* cause reduced yields

**Figure 1:**
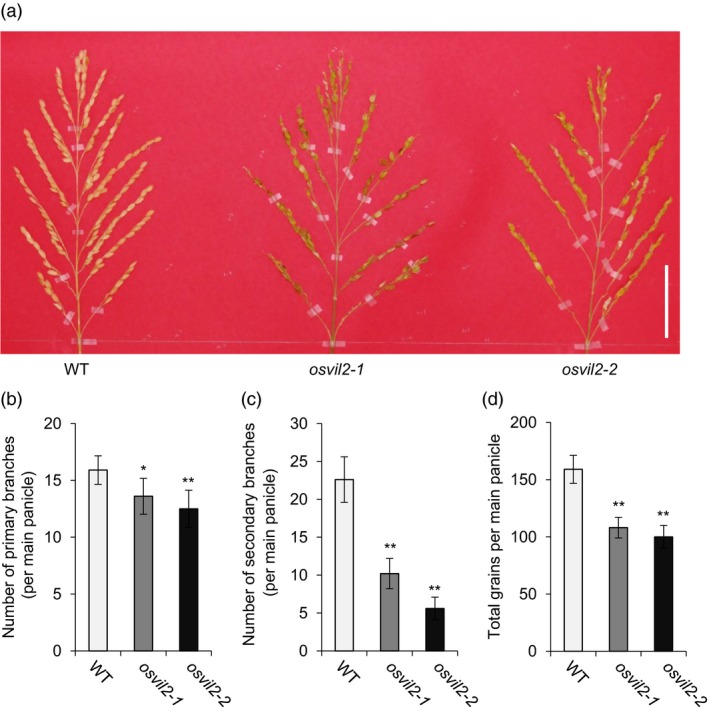
Phenotypes of WT and *osvil2* mutants. (a) Comparison of panicles among WT,* osvil2‐1*, and *osvil2‐2*. Scale bar = 5 cm. (b) Number of primary branches on main panicle. (c) Number of secondary branches on main panicle. (d) Number of grains from main panicle. Error bars show standard deviations; *n *=* *10. Statistical significance is indicated by * (*P *<* *0.01) and ** (*P *<* *0.001).

We previously described how mutations in OsVIL2 cause late flowering under both short‐ and long‐day conditions (Yang *et al*., 2013). Here, we observed additional phenotypes from two T‐DNA insertion mutants, *osvil2‐1* and *osvil2‐2*. In both mutant lines, grain number was reduced due to a decrease in primary and secondary branch numbers (Figure 1a‐c). This resulted in yield reductions of 32.0% (*osvil2‐1*) and 37.1% (*osvil12‐2*) (Figure 1d). In addition, the mutants displayed abnormal floral organ development that caused low fertility. The presence of these phenotypes suggested that OsVIL2 functions in controlling rice architecture and grain yield.

### *OsVIL2* OX plants exhibit phenotypes of increased yields

**Figure 2:**
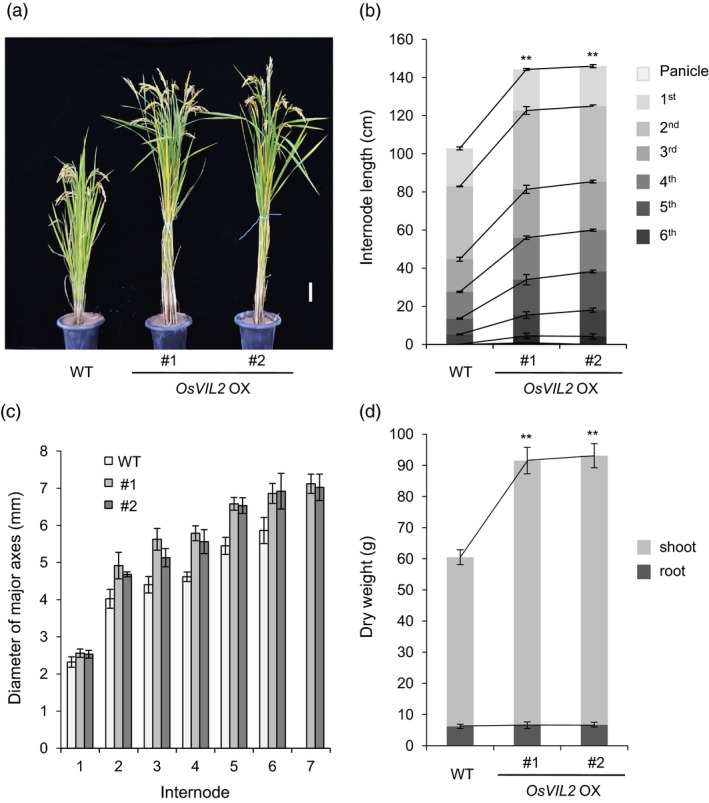
Morphological comparison between wild‐type (WT) and *OsVIL2*‐overexpression (OX) plants #1 and #2. (a) Phenotypes at seed‐ripening stage. Scale bar = 10 cm. (b) Lengths of panicles and internodes at seed‐ripening stage. (c) Diameter of each internode in major culm. (d) Comparison of dry weights among WT and *OsVIL2*‐OX plants at heading stage. Error bars indicate standard deviations; *n *=* *5 or more. Statistical significance is indicated by ** (*P *<* *0.001).

We generated transgenic plants (*OsVIL2*‐OX) that express *OsVIL2* cDNA under the maize *Ubi* promoter. From the 26 independent transformants, we selected two lines with higher expression of *OsVIL2* for further analyses. At the seed ripening stage, the OX plants were taller than segregating wild type (WT) (Figure 2a) because the transgenic plants had more and longer internodes (Figure 2b). The diameters of their major culms were also increased (Figure 2c). Total dry weight was also significantly increased in the OX plants when compared with the segregated WT (Figure 2d).

**Figure 3:**
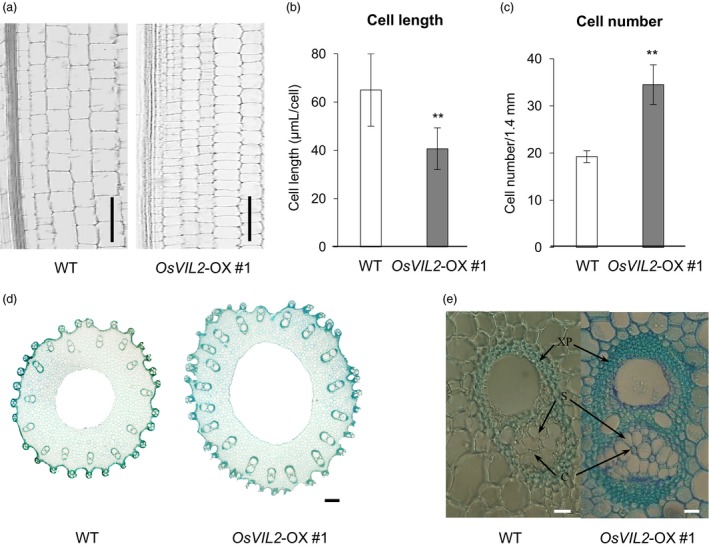
Longitudinal and cross sections of internode. (a) Longitudinal sections of culm from first internode of WT and *OsVIL2*‐OX #1 at heading stage. Scale bar: 100 μm. Comparison of cell length (b) and cell number (c) at about 0.5 cm upper part from the division region of the first internode of WT and *OsVIL2*‐OX #1 at heading stage. Error bars indicate standard deviations from four individual sections. Statistical significance is indicated by ** (*P *<* *0.001). (d) Cross sections of culm from first internode of WT and *OsVIL2*‐OX #1 plants. Scale bar = 200 μm. (e) Large vascular bundle from first internode. Arrows indicate xylem parenchyma cells (XP), sieve tubes (S) and companion cells (C). Scale bar = 500 μm.

To investigate whether the increase was due to change in cell number or cell size, the basal 0.5 cm region of the first internode was harvested at heading stage. The first internode is the most activity growing region at the stage and the basal region contains rapidly divining cells. Longitudinal sectioning of the dividing zone of the first internode showed that cells from the transgenic plants were reduced to 62.6% in length but increased to 172% in cell numbers when compared to WT (Figure 3a‐c). Cross sections of the region also indicated that culms from *OsVIL2*‐OX plants were thicker when compared with the segregated WT (Figure 3d). The OX plants also had more vascular bundles, sieve tubes and companion cells, as well as increased layers of xylem parenchyma cells (Figure 3d, e).

**Figure 4:**
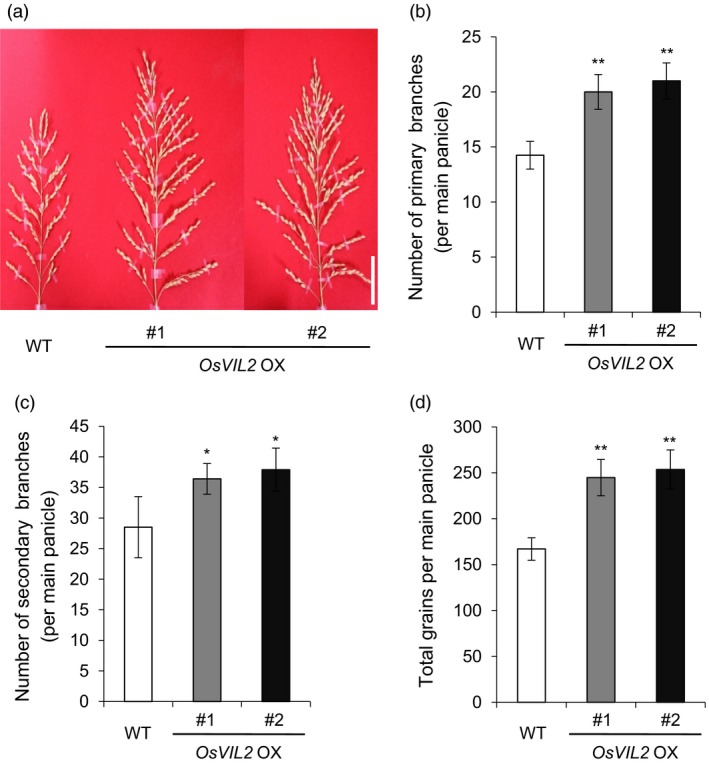
Panicle phenotypes. (a) Comparison between WT and two *OsVIL2 OX* transgenic lines. Scale bar = 5 cm. (b) Number of primary branches on main panicle. (c) Number of secondary branches on main panicle. (d) Number of grains from main panicle. Error bars show standard deviations; *n *=* *10. Statistical significance is indicated by * (*P *<* *0.01) and ** (*P *<* *0.001).

In addition, the *OsVIL2*‐OX plants developed larger panicles (Figure 4a) and more primary (Figure 4b) and secondary branches (Figure 4c) when compared with the WT. They also had 46.6% (*OsVIL2*‐OX #1) and 57.8% (*OsVIL2*‐OX #2) more grains in the main culm (Figure 4d). These *OsVIL2*‐OX phenotypes suggested that OsVIL2 functions to induce cell division and enhance meristem activity at the reproductive stages.

To study whether these phenotypes were reproducible when plants were grown in a large paddy field, we performed 2 years of tests at two different locations. Under field conditions, the same phenotype was maintained as observed in our small‐scale experiments, i.e., the OX plants were taller and had larger panicles than the WT. In the first test, grain numbers per panicle were increased by 29.6% and 34.8% in *OsVIL2*‐OX #1 and *OsVIL2*‐OX #2, respectively, and total yield was increased by 28.1%–32.2% over that measured from the WT, although tiller numbers were slightly decreased in the OX plants (Table 1). In the second trial at a different location, the OX plants produced 25.5% and 22.8% more grains per panicle compared with the WT, and total yield was increased to 118.0% and 115.8% of WT (Table 2).

**Table 1:** The first yield test of *OsVIL2*‐OX plants

Traits	Grains per panicle	Panicles per plant	Grains per plant
WT	92.9 ± 1.4 (100.0%)	13.6 ± 1.4 (100.0%)	1270.7 ± 47.7 (100.0%)
OsVIL2‐OX #1	120.4 ± 5.1 (129.6%)	12.9 ± 1.5 (94.8%)	1551.2 ± 34.9 (128.1%)
OsVIL2‐OX #2	125.3 ± 2.3 (134.8%)	12.6 ± 0.9 (92.6%)	1574.7 ± 32.3 (132.2%)

Plants were grown in paddy field at Gunwi, Korea (36°24′N) in 2014 at a spacing of 15 cm × 30 cm, with three plants per hill. The plot area was 5.4 m^2^. Values are means with standard deviations of three replications.

**Table 2:** The second yield test of *OsVIL2*‐OX plants

Traits	Grains per panicle	Panicles per plant	Grains per plant	Grain weight per plant	Fertility (%)
WT	136.7 ± 11.6 (100.0%)	14.7 ± 1.7 (100.0%)	1997.7 ± 148.4 (100.0%)	42.4 ± 3.1 (100.0%)	89.3 ± 5.1
OsVIL2‐OX #1	171.6 ± 20.5 (125.5%)	13.9 ± 1.5 (94.6%)	2356.9 ± 193.6 (118.0%)	52.1 ± 4.3 (122.9%)	86.0 ± 5.0
OsVIL2‐OX #2	167.8 ± 13.3 (122.8%)	13.8 ± 1.5 (93.9%)	2314.3 ± 158.0 (115.8%)	50.7 ± 3.5 (119.6%)	85.6 ± 3.5

Plants were grown in paddy field at Yongin, Korea (37°14′N) in 2017 at a spacing of 30 cm × 30 cm, with three plants per hill. The plot area was 9 m^2^. Values are means with standard deviations of three replications.

### Transcriptome analyses of *OsVIL2‐*OX plants

To determine which genes enhance cell division in the *OsVIL2*‐OX plants, we performed transcriptome analyses using mRNAs prepared from the 0.5 cm basal region of the first internodes sampled from *OsVIL2*‐OX #1 and WT plants at the heading stage. This region is highly meristematic at the stage. Using results from the RNA sequencing analysis, we identified 27 801 annotated genes, among which 1958 had at least twofold higher transcript levels (Table S1) while 2096 had at least twofold lower levels in *OsVIL2*‐OX than in the WT and (Table S2). They can be classified into 20 functional groups by MapMan analysis (Tables S3, S4 and S5). Genes in functional groups of cell division/cell cycle, cell organization, DNA synthesis and protein synthesis/amino acid activation were significantly abundant in the increased genes from *OsVIL2*‐OX plants (Table S3).

Because PHD‐PRC2 complex represses target gene expression (De Lucia *et al*., 2008; Wood *et al*., 2006), we suspected that transcript levels for the direct targets of our histone‐binding gene would be reduced in the *OsVIL2*‐OX plants. We therefore selected down‐regulated genes for verifying the results of the RNA sequencing experiment. From that group of genes, we randomly selected four TF genes (LOC_Os01g15900, LOC_Os03g02550, LOC_Os06g19444 and LOC_Os12g03040) and two chromatin interacting factor genes (LOC_Os02g58160 and LOC_Os11g15040). Quantitative real‐time RT‐PCR analyses confirmed that all were down‐regulated in the *OsVIL2*‐OX plants (Figure S1).

### OsVIL2 directly regulates the expression of *OsCKX2*

**Figure 5:**
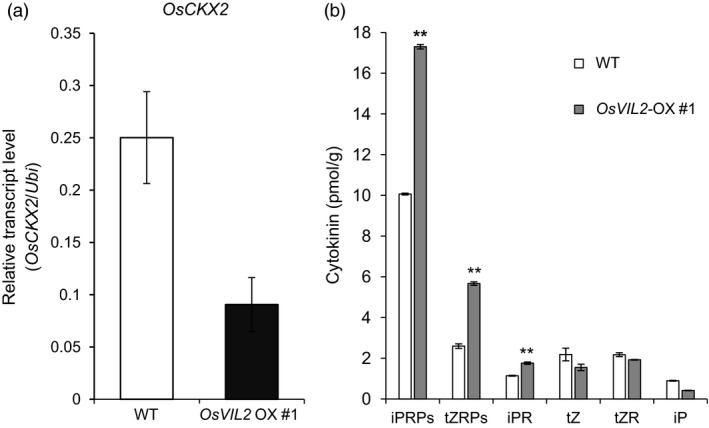
*OsCKX2* transcription and cytokinin concentrations in *OsVIL2*‐OX, mutant and WT plants. (a) Transcript levels in first internodes from WT and *OsVIL2*‐OX #1 were measured by quantitative real‐time PCR. *Y*‐axis, relative transcript level of *OsCKX2* compared with that of *Ubi*. Error bars indicate standard deviations; *n* = 3 or more. (b) Cytokinin levels in first internodes of WT and *OsVIL2*‐OX #1. iPRPs, N6‐(Δ2‐isopentenyl) adenine ribotides; tZRPs, trans‐zeatin ribotides; iPR, N6‐(Δ2‐isopentenyl) adenine riboside; tZ, trans‐zeatin; tZR, trans‐zeatin riboside; iP, N6‐(Δ2‐isopentenyl) adenine. Error bars indicate standard deviations; *n *=* *3 or more. Statistical significance is indicated by ** (*P *<* *0.001).

Cytokinin regulates the size of reproductive meristems and number of seeds by activating cell division and differentiation (Ashikari *et al*., 2005; Jameson and Song, 2016; Kyozuka, 2007). Because *OsVIL2*‐OX plants exhibited phenotypes of increased grain yield and biomass, we speculated that the phenotypes might be due to elevated cytokinin levels. Interestingly, expression levels of *OsCKX2* (LOC_Os01g10110) and *OsCKX4* (LOC_Os01g71310), that encode cytokinin‐degrading enzymes, were decreased in the *OsVIL2*‐OX plants (Table S2). Because *OsCKX2* is a major QTL that controls grain number (Ashikari *et al*., 2005), we selected *OsCKX2* for further analysis as a potential target of OsVIL2. Quantitative real‐time RT‐PCR analyses confirmed that the *OsCKX2* transcript level was much lower in *OsVIL2*‐OX plants (Figure 5a). Although *LP*,* DST* and *DEP1* also regulate grain yield (Huang *et al*., 2009; Li *et al*., 2011, 2013), we did not analyze them because their transcription was not altered in the OX plants (Figure S2).

Consistent with the role of OsCKX2 in reducing the amount of active cytokinins (Ashikari *et al*., 2005; Li *et al*., 2013), we observed that levels of active cytokinins, i.e., N6‐(Δ2‐isopentenyl) adenine riboside (iPR), N6‐(Δ2‐isopentenyl) adenine ribotides (iPRPS) and *trans*‐zeatin ribotides (tZRPs), were higher in the OX plants than in the WT (Figure 5b).

**Figure 6:**
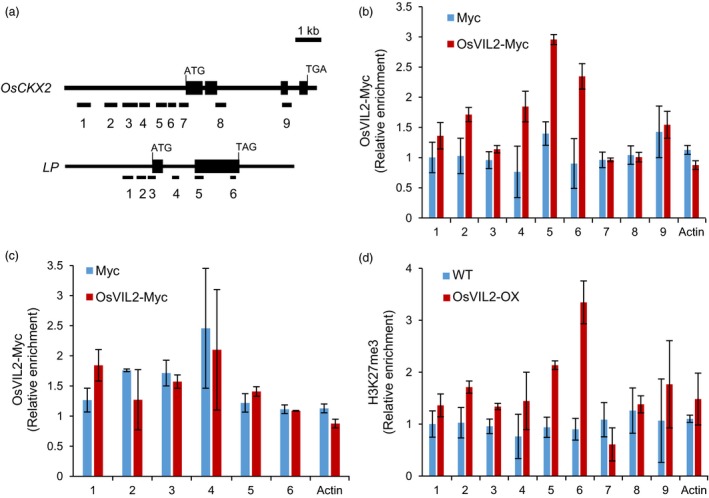
Chromatin immunoprecipitation assay of *OsCKX2* chromatin with OsVIL2‐Myc. (a) Genomic structure of *OsCKX2* and *OsLP*. Tested regions are numbered. (b) ChIP analysis of OsVIL2 enrichment on *OsCKX2* chromatin. OsVIL2‐Myc epitope‐tagged transgenic lines were used to detect enrichment. *Actin* chromatin served as control. Samples from OsVIL2‐Myc plants are indicated in red, while those from control plants expressing only Myc are in blue. (c) ChIP analysis of OsVIL2 enrichment on *OsLP1* chromatin. OsVIL2‐Myc epitope‐tagged transgenic lines were used to detect enrichment. *Actin* chromatin served as control. Samples from OsVIL2‐Myc plants are indicated in red, while those from control plants expressing only Myc are in blue. (d) Analysis of H3K27me3 level on *OsCKX2* chromatin in WT (blue) and *OsVIL2*‐OX #1 (red) using antibodies against H3K27me3. *Actin* chromatin served as control. Error bars show standard deviations; *n *=* *3.

To examine whether OsVIL2 directly regulates *OsCKX2* expression, we performed chromatin immunoprecipitation (ChIP) assays using transgenic plants that express Myc‐tagged OsVIL2 protein. Transgenic plants expressing Myc alone were used as the control. Assays using anti‐Myc antibody revealed an enrichment of OsVIL2 at the transcript start region of *OsCKX2* (Figure 6a, b). As a negative control, we used *OsLP* and found that it was not enriched in the OsVIL2‐Myc transgenic plants (Figure 6c).

Because PHD‐PRC2 complex can increase the H3K27me3 levels of target loci (Sung and Amasino, 2004; Sung *et al*., 2006; Yang *et al*., 2013), we measured the level of H3K27me3 in *OsCKX2* chromatin using H3K27me3 antibodies. In the *OsVIL2*‐OX plants, *OsCKX2* chromatin was significantly enriched by the antibodies at the region near the transcript start site (Figure 6d). This result suggested that *OsCKX2* expression was reduced in those OX plants by mediating methylation of the H3K27 in its promoter regions.

### OsVIL2 interacts with OsEMF2b through the FNIII domain

**Figure 7:**
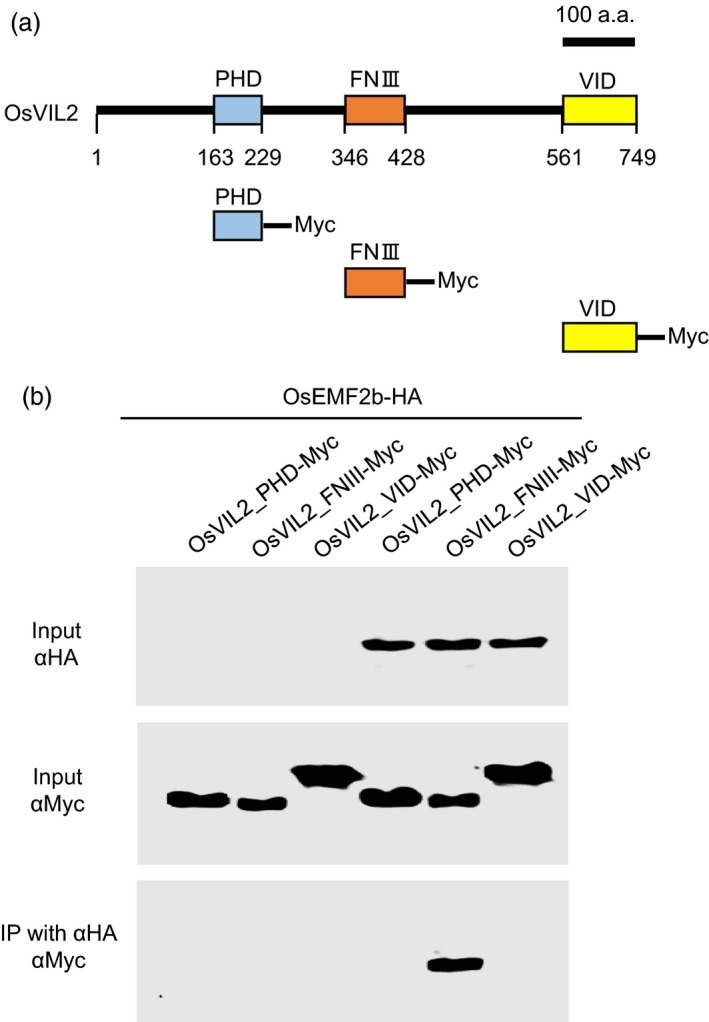
Analysis of interaction between OsVIL2 and OsEMF2b by co‐immunoprecipitation assay. (a) Schematic representation of OsVIL2 protein. Scale bar = 100 a.a. (b) Co‐immunoprecipitation assay between OsEMF2b and 3 domains of OsVIL2. Total proteins were extracted from Oc‐cell protoplasts after transient expression of OsVIL2_PHD‐Myc, OsVIL2_FNIII‐Myc, OsVIL2_VID‐Myc and OsEMF2b‐HA. Extracts were immuno‐precipitated with anti‐HA antibodies and interaction signals were detected using anti‐Myc antibody after SDS‐PAGE. Inputs, total protein extracts before immunoprecipitation; IP, elutes from agarose beads after immunoprecipitation. The entire experiment was conducted three times.

We have reported previously that OsVIL2 binds to OsEMF2b, a core component of PRC2 complex (Yang *et al*., 2013). To investigate the region that binds to OsEMF2b, we sub‐cloned three conservative motifs in OsVIL2 and fused them to Myc (Figure 7a). Interactions between OsEMF2b and the OsVIL2 fragments were analyzed via co‐immunoprecipitation (Co‐IP) assays. The analyses showed that the fragment containing FNIII domain binds to OsEMF2b, while the other fragments carrying either the PHD or VID domain did not interact with the PRC2 subunit (Figure 7b).

## Discussion

### OsVIL2 represses *OsCKX2* expression by chromatin modulating

Although several genes that regulate grain yield have been identified, little is known about how they are modulated by chromatin remodelling. In this study, we determined that OsVIL2 controls grain yield through the chromatin remodelling of *OsCKX2*. OsVIL2 is highly homologous to Arabidopsis VIN3 and VILs, which form PHD‐PRC2 complexes. Moreover, the conserved motifs of the PHD finger domain, FNIII domain and VID found in Arabidopsis VILs are also present in rice VILs (Greb *et al*., 2007; Sung *et al*., 2006).

We observed that OsVIL2 binds to histone H3, supporting its role as a chromatin interacting factor (Yang *et al*., 2013). We also showed that OsVIL2 binds to the FNIII domain of OsEMF2b, which is a homologue of *Drosophila* Su(z)12 and a core member of PRC2 (Hennig and Derkacheva, 2009). In Arabidopsis, the complex between VILs and PRC2 core proteins provides the trimethylation activity to target chromatins and influences vegetative development, flowering time and floral organ development (Conrad *et al*., 2014; Schonrock *et al*., 2006; Yang *et al*., 2013; Yoshida *et al*., 2001). Because OsVIL2 also binds to EMF2b and histone H3, it is likely that the former forms a complex with core PRC2 via EMF2b. This complex would function in histone methylation because the level of H3K27me3 in the *OsCKX2* promoter region was changed in the OsVIL2‐OX plants. Further studies are needed to elucidate molecular mechanisms how the OsVIL2‐PRC2 complex associates to the target chromatin in rice.

### OsVIL2 enhances grain yield by increasing cytokinin concentrations

The balance between cytokinin synthesis and catabolism is an important element in controlling meristem activity and grain yield (Mok and Mok, 2001; Sakakibara, 2006). The CKX protein has a critical role in controlling cytokinin levels in the shoot apical meristem (SAM). For example, overexpression of *CKX3* retards the formation of leaf and flower primordia in *Arabidopsis* (Werner *et al*., 2003). In contrast, *ckx3* mutation enhances cytokinin concentrations in SAM and enlarges the meristem (Bartrina *et al*., 2011).

In rice, *LONELY GUY* (*LOG*) encodes a phosphoribohydrolase that converts cytokinin nucleotides to free‐base forms such as iPR and tZR, which are active cytokinins. The *log* mutants have fewer branches and spikelets (Kurakawa *et al*., 2007). By contrast, a reduction in *OsCKX2* expression results in the accumulation of active cytokinin and a greater number of spikelets, suggesting that increases in endogenous cytokinin levels in the inflorescences causes them to form large meristems (Ashikari *et al*., 2005). In this study, we showed that the chromatin interacting factor OsVIL2 elevates cytokinin levels by suppressing *OsCKX2* expression. Activated expression of *OsCKX4* reduces cytokinin levels, and those plants are shorter and produce less panicle branches and grains (Gao *et al*., 2014). We did not study *OsCKX4* because it has a major role in modulating crown root development (Gao *et al*., 2014). However, we do not rule out a possibility that *OsCKX4* is also a target of OsVIL2.

Cytokinin promotes cell division and proliferation. We showed that cell numbers were dramatically increased at the basal part of the first internode in *OsVIL2*‐OX plants compared with the WT. RNA sequencing analysis of the basal part revealed that expression levels of a large number of genes that function in cell division and cell cycles, as well as those involved in synthesis of DNA, and protein were elevated in the *OsVIL2*‐OX plants. This observation suggests that elevated level of cytokinins in the *OsVIL2*‐OX plants enhanced meristem activity and cell division that resulted in increase in plant biomass and yield.

The RNA sequencing analysis also revealed that *WFP* (LOC_Os08g39890) and *TERMINAL FLOWER 1* (*TFL1*)/*CENTRORADIALIS* (*CEN*)‐like genes (*RCN1*; LOC_Os11g05470 and *RCN2*; LOC_Os02g32950) were up‐regulated in the *OsVIL2*‐OX plants. In previous studies, overexpression of *WFP* increases total grain number per panicle by producing more primary branches (Miura *et al*., 2010). However, overexpression of *RCN1* increases grain productivity by producing more secondary and tertiary branches rather than primary branches (Nakagawa *et al*., 2002). In the *OsVIL2*‐OX plants, the number of both primary and secondary branches was increased compared with the WT. It has been suggested that fine‐tuning of *SPL* and *RCN* regulates vegetative and reproductive branching in rice (Wang *et al*., 2015). Therefore, up‐regulation of these genes in the *OsVIL2*‐OX plants might play an important role in increasing total grain yield by modulating the activities of inflorescence and branch meristems.

### OsVIL2 modulates plant architecture

To increase productivity, breeders have been selecting semi‐dwarf varieties that grow well without chemical fertilizers (Asano *et al*., 2007; Ashikari *et al*., 2002). Tall plants generally are not favoured because they are susceptible to lodging. However, tall, sturdy stems and fewer tillers are considered important characteristics of ideal plant architecture, or IPA (Khush, 1995; Virk *et al*., 2004). Both *IPA1* and *WFP* contribute to IPA and increased grain yield (Jiao *et al*., 2010; Miura *et al*., 2010). Rice cultivars containing *IPA1*/*WFP* are tall and have fewer tillers but more branches and spikelets. Their thicker culms also improve their resistance to lodging (Jiao *et al*., 2010; Miura *et al*., 2010). Another effective QTL is *STRONG CULM2* (*SCM2*) (Ookawa *et al*., 2010). *SCM2*‐carrying cultivars have sturdier culms and more spikelets that lead to higher yields and better lodging resistance (Ookawa *et al*., 2010). We also demonstrated here that increased expression of *OsVIL2* results in a phenotype of tall, thicker culms, greater branching and higher grain numbers. Therefore, this gene could be used for achieving IPA in molecular breeding programmes.

## Experimental procedures

### Plant materials and growth

The T‐DNA mutant lines were isolated from a T‐DNA tagging line (*Oryza sativa* japonica cv. Dongjin and Hwayoung) (Jeon *et al*., 2000; Jeong *et al*., 2002). Their flanking sequences were determined by inverse PCR (An *et al*., 2003; Jeong *et al*., 2006; Ryu *et al*., 2004). We previously described the T‐DNA insertional mutants *osvil2‐1* and *osvil2‐2* as well as transgenic plants that express OsVIL2‐Myc (Yang *et al*., 2013). All plants were grown either in the greenhouse or in certified genetically modified organism (GMO) fields at Yongin and Gunwi, Korea. For the field tests, three seedlings were planted per hill, at a spacing of 15 × 30 cm, as reported previously (Lee and An, 2015). Whole plants including roots were harvested at heading stage from the paddy. Roots were carefully washed to remove the soil. Plants were dried at 65 °C for 5 d before measuring dry weight.

### Vector construction and plant transformation

The full‐length *OsVIL2* cDNA clone was isolated by PCR, using two primers: 5′‐AAGCTTCAATTCGCCATG GATCCACC‐3′ and 5′‐ACTAGTATGCCAAAGT TCCATGCA‐3′. An amplified fragment was digested with restriction enzymes *Hind*III and *Spe*I, and inserted into the pGA3426 vector under the control of the maize *ubiquitin 1* promoter (Kim *et al*., 2009). The construct was then transferred into *Agrobacterium tumefaciens* LBA4404 by the freeze‐thaw method (An *et al*., 1988). Procedures for transforming rice via *Agrobacterium*‐mediated co‐cultivation were described previously (Jeon *et al*., 1999; Yoon *et al*., 2014).

### RNA extraction and RT‐PCR analyses

Total RNA was isolated using RNAiso Plus (Takara, Shiga, Japan; http://www.takara-bio.com). First‐strand cDNA was synthesized with 2 μg of total RNA and Moloney murine leukaemia virus reverse transcriptase (Promega, Madison, WI, USA; http://www.promega.com). Synthesized cDNA was prepared from leaf blades, and quantitative real‐time RT‐PCR was performed with a Rotor‐Gene 6000 (Corbett Research, Sydney, Australia; http://www.corbettlifescience.com), following protocols reported earlier (Cho *et al*., 2016; Yang *et al*., 2013). Rice *OsUbi1* was used as an internal control. Primers for studying gene expression are listed in Table S6.

### Transcriptome analysis

Total RNA was extracted from the 0.5 cm basal region of the first internodes of WT and *OsVIL2*‐OX #1 plants at heading stage in duplicate. This region contains meristematic activity. The mRNA of each sample was enriched using oligo(dT) magnetic beads and digested into short fragments by fragmentation buffer. Random hexamers were used as primers for first‐strand and second‐strand cDNA synthesis. These double‐stranded samples were treated with T4 DNA polymerase and T4 polynucleotide kinase for end‐repairing and dA‐tailing, followed by T4 DNA ligase treatment for adaptor ligation. Afterward, fragments approximately 200 bp long were collected and used as templates for PCR amplification to create the cDNA library. This library was pair‐end sequenced using the PE90 strategy (paired‐end reads of 90 base pairs per read) on an Illumina HiSeq™ 2000 at the Beijing Genomics Institute (Wuhan, China). The raw reads were processed to generate clean‐read datasets by removing the adaptor sequences, reads with >5% ambiguous bases (noted as N), and low‐quality reads that contained more than 20% of bases with qualities of <20. The clean reads were then aligned to the rice japonica genome (version: Tigr 7.0) using the Tophat program (v2.0.11) under the following parameters: ‐a 10 ‐m 0 ‐i 31 ‐I 500000 –G (Trapnell *et al*., 2012). The DEseq algorithm was applied to filter the differentially expressed genes. Genes with significantly different expression were determined by FDR < 0.05 and fold changes of >2 or <0.5 in two samples (Anders and Huber, 2010).

### GO analysis

The GO information of differently expressed genes were retrieved from the rice oligonucleotide array database (now available at http://ricephylogenomics-khu.org/ROAD/analysis/go_enrichment.shtml.) (Cao *et al*., 2012). Fold‐enrichment values were calculated by dividing the query number by the query expected value. We selected GO terms with a fold‐enrichment greater than 2 and a hyper‐geometric *P*‐value below 0.05. Visualization of GO terms was done by EXCEL software, and Illustrator software was used to polish GO terms. Functional information of differentially expressed genes was analyzed by the MapMan toolkit (3.6.0RC1), which has been frequently used for functional classification of transcriptome data (Thimm *et al*., 2004).

### Measurements of cytokinin levels

Extractions and determinations of cytokinins from the division region of the first internodes of the WT and *OsVIL2*‐OX #1 plants were performed with an UPLC‐MS/MS (AQITY UPLC™ System/Quattro Ultima Pt; Waters) and ODS column (AQUITY UPLC BEH C18, 1.7 μm, 2.1 × 100 mm; Waters), as described previously (Kojima *et al*., 2009).

### Co‐immunoprecipitation assay

The Co‐IP assays were performed as reported earlier (Cho *et al*., 2016; Yoon *et al*., 2017). Briefly, fusion molecules were co‐expressed in rice Oc cell protoplasts. Afterwards, fusion proteins were extracted in IP buffer [75 mm NaCl, 50 mm Tris‐HCl (pH 7.5), 5 mm EDTA, 1% Triton X‐100, 1 mm dithiothreitol, 1 mm phenylmethanesulfonylfluoride, 2 mm NaF, 20 μm MG132, and an appropriate amount of Protease inhibitor cocktail (Roche)]. Expressed proteins were immuno‐precipitated with anti‐HA mouse monoclonal antibodies (12CA5; Roche, Mannheim, Germany, http://www.roche.com) conjugated with A and G agarose beads (Millipore, Billerica, MA, USA, http://www.emdmillipore.com). For protein detection, we used horseradish peroxidase (HRP)‐conjugated anti‐Myc monoclonal antibody (#2040; Cell Signaling) and anti‐HA monoclonal antibody (#2999; Cell Signaling).

### Chromatin immunoprecipitation analysis

Transgenic plants expressing OsVIL2‐Myc were used for ChIP analysis, as described previously (Yang *et al*., 2013; Yoon *et al*., 2017), with the anti‐Myc monoclonal antibody (Cell Signaling; #2276) and the anti‐H3K27me3 monoclonal antibody (Millipore; 07‐449). The assays were performed as reported earlier (Haring *et al*., 2007). Primer sequences are listed in Table S6. All assays were performed at least three times from two biological replicates.

### Statistical analyses

The *P* values were calculated using one‐way analysis of variance (ANOVA; Tukey HSD test) for the test groups with R program (Cohen and Cohen, 2008).

## Conflict of interest

The authors declare they have no conflict of interest.

## Supporting information


**Figure S1** Confirmation of RNA sequencing by quantitative real‐time PCR.
**Figure S2** Transcript levels of *LP*,* DST* and *DEP1* in WT and *OsVIL2*‐OX plants.

**Table S1** Differentially up‐regulated genes in *OsVIL2*‐OX**.**

**Table S2** Differentially down‐regulated genes in *OsVIL2*‐OX.

**Table S4** MapMan analysis of up‐regulated genes in *OsVIL2*‐OX**.**

**Table S5** MapMan analysis of down‐regulated genes in *OsVIL2*‐OX**.**

**Table S3** Classification of up‐ and down‐regulated genes in *OsVIL2*‐OX. The functional groups that are significantly abundant in the increased genes are indicated in blue.
**Table S6** Primers used in this study.

## References

[pbi12956-bib-0001] An, G. , Ebert, P.R. , Mitra, A. and Ha, S.B. (1988) Binary vectors In Plant Molecular Biology Manual (Gelvin S.B. and Schilperoort R.A. eds), pp. 1–19. Dordrecht: Kluwer Academic Publisher. A3.

[pbi12956-bib-0002] An, S. , Park, S. , Jeong, D.H. , Lee, D.Y. , Kang, H.G. , Yu, J.H. , Hue, J. *et al* (2003) Generation and analysis of end sequence database for T‐DNA tagging lines in rice. Plant Physiol. 133, 2040–2047. 14630961 10.1104/pp.103.030478 PMC300755

[pbi12956-bib-0003] Anders, S. and Huber, W. (2010) Differential expression analysis for sequence count data. Genome Biol. 11, R106. 20979621 10.1186/gb-2010-11-10-r106 PMC3218662

[pbi12956-bib-0004] Asano, K. , Takashi, T. , Miura, K. , Qian, Q. , Kitano, H. , Matsuoka, M. and Ashikari, M. (2007) Genetics and molecular analysis of utility of *sd1* alleles in rice breeding. Breed. Sci. 57, 53–58.

[pbi12956-bib-0005] Ashikari, M. , Sasaki, A. , Ueguchi‐Tanaka, M. , Itoh, H. , Nishimura, A. , Datta, S. , Ishiyama, K. *et al* (2002) Loss‐of‐function of a rice gibberellin biosynthetic gene, *GA20 oxidase* (*GA20ox‐2*), led to the rice ‘green revolution’. Breed. Sci. 52, 143–150.

[pbi12956-bib-0006] Ashikari, M. , Sakakibara, H. , Lin, S. , Yamamoto, T. , Takashi, T. , Nishimura, A. , Angeles, E.R. *et al* (2005) Cytokinin oxidase regulates rice grain production. Science, 309, 741–745. 15976269 10.1126/science.1113373

[pbi12956-bib-0007] Bartrina, I. , Otto, E. , Strnad, M. , Werner, T. and Schmulling, T. (2011) Cytokinin regulates the activity of reproductive meristems, flower organ size, ovule formation, and thus seed yield in *Arabidopsis thaliana* . Plant Cell, 23, 69–80. 21224426 10.1105/tpc.110.079079 PMC3051259

[pbi12956-bib-0008] Cao, R. , Wang, L. , Wang, H. , Xia, L. , Erdjument‐Bromage, H. , Tempst, P. , Jones, R.S. *et al* (2002) Role of histone H3 lysine 27 methylation in Polycomb‐group silencing. Science, 298, 1039–1043. 12351676 10.1126/science.1076997

[pbi12956-bib-0009] Cao, P. , Jung, K.H. , Choi, D. , Hwang, D. , Zhu, J. and Ronald, P.C. (2012) The rice oligonucleotide array database: An atlas of rice gene expression. Rice, 5, 17. 24279809 10.1186/1939-8433-5-17 PMC4883718

[pbi12956-bib-0011] Cho, L.H. , Yoon, J. , Pasriga, R. and An, G. (2016) Homodimerization of Ehd1 is required to induce flowering in rice. Plant Physiol. 170, 2159–2171. 26864016 10.1104/pp.15.01723 PMC4825144

[pbi12956-bib-0012] Cohen, Y. and Cohen, J.Y. (2008) Analysis of Variance, in Statistics and Data with R: An Applied Approach through Examples. Chichester, UK: John Willey & Sons Ltd 10.1002/9780470721896.

[pbi12956-bib-0013] Conrad, L.J. , Khanday, I. , Johnson, C. , Guiderdoni, E. , An, G. , Vijayraghavan, U. and Sundaresan, V. (2014) The polycomb group gene *EMF2B* is essential for maintenance of floral meristem determinacy in rice. Plant J. 80, 883–894. 25279942 10.1111/tpj.12688

[pbi12956-bib-0014] De Lucia, F. , Crevillen, P. , Jones, A.M. , Greb, T. and Dean, C. (2008) A PHD‐polycomb repressive complex 2 triggers the epigenetic silencing of *FLC* during vernalization. Proc. Natl. Acad. Sci. USA, 105, 16831–16836. 18854416 10.1073/pnas.0808687105 PMC2579339

[pbi12956-bib-0015] Fu, D. , Dunbar, M. and Dubcovsky, J. (2007) Wheat VIN3‐like PHD finger genes are up‐regulated by vernalization. Mol. Genet. Genom. 277, 301–313. 10.1007/s00438-006-0189-6 17123111

[pbi12956-bib-0016] Gao, S. , Fang, J. , Xu, F. , Qang, W. , Sun, X. , Vhu, J. , Vai, B. *et al* (2014) Cytokinin oxidase/dehydrogenase gene *OsCKX4* integrates cytokinin and auxin signaling to control rice crown root formation. Plant Physiol. 165, 1035–1046. 24808099 10.1104/pp.114.238584 PMC4081320

[pbi12956-bib-0017] Greb, T. , Mylne, J.S. , Crevillen, P. , Geraldo, N. , An, H. , Gendall, A.R. and Dean, C. (2007) The PHD finger protein VRN5 functions in the epigenetic silencing of *Arabidopsis FLC* . Curr. Biol. 17, 73–78. 17174094 10.1016/j.cub.2006.11.052

[pbi12956-bib-0018] Haring, M. , Offermann, S. , Danker, T. , Horst, I. , Peterhansel, C. and Stam, M. (2007) Chromatin immunoprecipitation: Optimization, quantitative analysis and data normalization. Plant Meth. 3, 1–16. 10.1186/1746-4811-3-11 PMC2077865 17892552

[pbi12956-bib-0019] Hennig, L. and Derkacheva, M. (2009) Diversity of Polycomb group complexes in plants: Same rules, different players? Trends Genet. 25, 414–423. 19716619 10.1016/j.tig.2009.07.002

[pbi12956-bib-0020] Huang, X.Z. , Qian, Q. , Liu, Z.B. , Sun, H.Y. , He, S.Y. , Luo, D. , Xia, G.M. *et al* (2009) Natural variation at the *DEP1* locus enhances grain yield in rice. Nat. Genet. 41, 494–497. 19305410 10.1038/ng.352

[pbi12956-bib-0021] Ikeda‐Kawakatsu, K. , Yasuno, H. , Oikawa, T. , Iida, S. , Nagato, Y. , Maekawa, M. and Kyozuka, J. (2009) Expression level of *ABERRANT PANICLE ORGANIZATION1* determines rice inflorescence form through control of cell proliferation in the meristem. Plant Physiol. 150, 736–747. 19386809 10.1104/pp.109.136739 PMC2689948

[pbi12956-bib-0022] Jameson, P.E. and Song, J. (2016) Cytokinin: A key driver of seed yield. J. Exp. Bot. 67, 593–606. 26525061 10.1093/jxb/erv461

[pbi12956-bib-0023] Jeon, J.S. , Chung, Y.Y. , Lee, S. , Yi, G.H. , Oh, B.G. and An, G. (1999) Isolation and characterization of an anther‐specific gene, RA8, from rice (*Oryza sativa L*.). Plant Mol. Biol. 39, 35–44. 10080707 10.1023/a:1006157603096

[pbi12956-bib-0024] Jeon, J.S. , Lee, S. , Jung, K.H. , Jun, S.H. , Jeong, D.H. , Lee, J. , Kim, C. *et al* (2000) T‐DNA insertional mutagenesis for functional genomics in rice. Plant J. 22, 471–570. 10886776 10.1046/j.1365-313x.2000.00767.x

[pbi12956-bib-0025] Jeong, D.H. , An, S. , Kang, H.G. , Moon, S. , Han, J.J. , Park, S. , Lee, H.S. *et al* (2002) T‐DNA insertional mutagenesis for activation tagging in rice. Plant Physiol. 130, 1636–1644. 12481047 10.1104/pp.014357 PMC166679

[pbi12956-bib-0026] Jeong, D.H. , An, S. , Park, S. , Kang, H.G. , Park, G.G. , Kim, S.R. , Sim, J. *et al* (2006) Generation of a flanking sequence tag database for activation‐tagging lines in japonica rice. Plant J. 45, 123–132. 16367959 10.1111/j.1365-313X.2005.02610.x

[pbi12956-bib-0027] Jeong, H.J. , Yang, H. , Yi, J. and An, G. (2015) Controlling flowering time by histone methylation and acetylation in *Arabidopsis* and rice. J. Plant Biol. 58, 203–210.

[pbi12956-bib-0028] Jiao, Y. , Wang, Y. , Xue, D. , Wang, J. , Yan, M. , Liu, G. , Dong, G. *et al* (2010) Regulation of *OsSPL14* by OsmiR156 defines ideal plant architecture in rice. Nat. Genet. 42, 541–544. 20495565 10.1038/ng.591

[pbi12956-bib-0029] Khush, G.S. (1995) Breaking the yield frontier of rice. GeoJournal, 35, 329–332.

[pbi12956-bib-0030] Kim, S.R. , Lee, D.Y. , Yang, J.I. , Moon, S. and An, G. (2009) Cloning vectors for rice. J. Plant Biol. 52, 73–78.

[pbi12956-bib-0031] Kohler, C. and Villar, C.B. (2008) Programming of gene expression by Polycomb group proteins. Trends Cell Biol. 18, 236–243. 18375123 10.1016/j.tcb.2008.02.005

[pbi12956-bib-0032] Kojima, M. , Kamada‐Nobusada, T. , Komatsu, H. , Takei, K. , Kuroha, T. , Mizutani, M. , Ashikari, M. *et al* (2009) Highly sensitive and high‐throughput analysis of plant hormones using MS‐probe modification and liquid chromatography‐tandem mass spectrometry: An application for hormone profiling in *Oryza sativa* . Plant Cell Physiol. 50, 1201–1214. 19369275 10.1093/pcp/pcp057 PMC2709547

[pbi12956-bib-0033] Komatsu, K. , Maekawa, M. , Ujiie, S. , Satake, Y. , Furutani, I. , Okamoto, H. , Shimamoto, K. *et al* (2003) *LAX* and *SPA*: Major regulators of shoot branching in rice. Proc. Natl. Acad. Sci. USA, 100, 11765–11770. 13130077 10.1073/pnas.1932414100 PMC208832

[pbi12956-bib-0034] Kurakawa, T. , Ueda, N. , Maekawa, M. , Kobayashi, K. , Kojima, M. , Nagato, Y. , Sakakibara, H. *et al* (2007) Direct control of shoot meristem activity by a cytokinin‐activating enzyme. Nature, 445, 652–655. 17287810 10.1038/nature05504

[pbi12956-bib-0035] Kyozuka, J. (2007) Control of shoot and root meristem function by cytokinin. Curr. Opin. Plant Biol. 10, 442–446. 17904411 10.1016/j.pbi.2007.08.010

[pbi12956-bib-0036] Lee, Y.S. and An, G. (2015) *OsGI* controls flowering time by modulating rhythmic flowering time regulators preferentially under short day in rice. J. Plant Biol. 58, 137–145.

[pbi12956-bib-0037] Li, M. , Tang, D. , Wang, K. , Wu, X. , Lu, L. , Yu, H. , Gu, M. *et al* (2011) Mutations in the F‐box gene *LARGER PANICLE* improve the panicle architecture and enhance the grain yield in rice. Plant Biotechnol. J. 9, 1002–1013. 21447055 10.1111/j.1467-7652.2011.00610.x

[pbi12956-bib-0038] Li, S. , Zhao, B. , Yuan, D. , Duan, M. , Qian, Q. , Tang, L. , Wang, B. *et al* (2013) Rice zinc finger protein DST enhances grain production through controlling *Gn1a*/*OsCKX2* expression. Proc. Natl. Acad. Sci. USA, 110, 3167–3172. 23382237 10.1073/pnas.1300359110 PMC3581943

[pbi12956-bib-0039] Luo, M. , Platten, D. , Chaudhury, A. , Peacock, W.J. and Dennis, E.S. (2009) Expression, imprinting, and evolution of the polycomb group genes. Mol. Plant, 2, 711–723. 19825651 10.1093/mp/ssp036

[pbi12956-bib-0040] Miura, K. , Ikeda, M. , Matsubara, A. , Song, X.J. , Ito, M. , Asano, K. , Matsuoka, M. *et al* (2010) *OsSPL14* promotes panicle branching and higher grain productivity in rice. Nat. Genet. 42, 545–549. 20495564 10.1038/ng.592

[pbi12956-bib-0041] Mok, D.W.S. and Mok, M.C. (2001) Cytokinin metabolism and action. Annu. Rev. Plant Physiol. 52, 89–118. 10.1146/annurev.arplant.52.1.89 11337393

[pbi12956-bib-0042] Nakagawa, M. , Shimamoto, K. and Kyozuka, J. (2002) Overexpression of *RCN1* and *RCN2*, rice *TERMINAL FLOWER 1/CENTRORADIALIS* homologs, confers delay of phase transition and altered panicle morphology in rice. Plant J. 29, 743–750. 12148532 10.1046/j.1365-313x.2002.01255.x

[pbi12956-bib-0043] Oikawa, T. and Kyozuka, J. (2009) Two‐step regulation of LAX PANICLE1 protein accumulation in axillary meristem formation in rice. Plant Cell, 21, 1095–1108. 19346465 10.1105/tpc.108.065425 PMC2685638

[pbi12956-bib-0044] Ookawa, T. , Hobo, T. , Yano, M. , Murata, K. , Ando, T. , Miura, H. , Asano, K. *et al* (2010) New approach for rice improvement using a pleiotropic QTL gene for lodging resistance and yield. Nat. Commun. 1, 132. 21119645 10.1038/ncomms1132 PMC3065348

[pbi12956-bib-0045] Ryu, C.‐H. , You, J.H. , Kang, H.G. , Hur, J. , Kim, Y.H. , Han, M.J. , An, K. *et al* (2004) Generation of T‐DNA tagging lines with a bidirectional gene trap vector and the establishment of an insertion‐site database. Plant Mol. Biol. 54, 489–502. 15316285 10.1023/B:PLAN.0000038257.93381.05

[pbi12956-bib-0046] Sakakibara, H. (2006) Cytokinins: Activity, biosynthesis, and translocation. Annu. Rev. Plant Biol. 57, 431–449. 16669769 10.1146/annurev.arplant.57.032905.105231

[pbi12956-bib-0047] Schonrock, N. , Bouveret, R. , Lewroy, O. , Borghi, L. , Kohler, C. , Gruissem, W. and Hennig, L. (2006) Polycomb‐group proteins repress the floral activator *AGL19* in the *FLC*‐independent vernalization pathway. Genes Dev. 20, 1667–1678. 16778081 10.1101/gad.377206 PMC1482485

[pbi12956-bib-0048] Schubert, D. , Clarenz, O. and Goodrich, J. (2005) Epigenetic control of plant development by Polycomb‐group proteins. Curr. Opin. Plant Biol. 8, 553–561. 16043386 10.1016/j.pbi.2005.07.005

[pbi12956-bib-0049] Schuettengruber, B. , Chourrout, D. , Vervoort, M. , Leblanc, B. and Cavalli, G. (2007) Genome regulation by polycomb and trithorax proteins. Cell, 128, 735–745. 17320510 10.1016/j.cell.2007.02.009

[pbi12956-bib-0050] Sung, S. and Amasino, R.M. (2004) Vernalization in *Arabidopsis thaliana* is mediated by the PHD finger protein VIN3. Nature, 427, 159–164. 14712276 10.1038/nature02195

[pbi12956-bib-0051] Sung, S. , Schmitz, R.J. and Amasino, R.M. (2006) A PHD finger protein involved in both the vernalization and photoperiod pathways in *Arabidopsis* . Genes Dev. 20, 3244–3248. 17114575 10.1101/gad.1493306 PMC1686601

[pbi12956-bib-0052] Thimm, O. , Bläsing, O. , Gibon, Y. , Nagel, A. , Meyer, S. , Krüger, P. , Selbig, J. *et al* (2004) MAPMAN: A user‐driven tool to display genomics data sets onto diagrams of metabolic pathways and other biological processes. Plant J. 37, 914–939. 14996223 10.1111/j.1365-313x.2004.02016.x

[pbi12956-bib-0530] Trapnell, C. , Roberts, A. , Goff, L. , Pertea, G. , Kim, D. , Kelly, D.R. , Pimentel, H. , *et al* (2012) Differential gene and transcript expression analysis of RNA‐seq experiments with TopHat and Cufflinks. Nat. Protoc. 7, 562–578. 22383036 10.1038/nprot.2012.016 PMC3334321

[pbi12956-bib-0053] Virk, P.S. , Khush, G.S. and Peng, S. (2004) Breeding to enhance yield potential of rice at IRRI: The ideotype approach. Int. Rice Res. Notes, 29, S1–S9.

[pbi12956-bib-0054] Wang, L. , Sun, S. , Jin, J. , Fu, D. , Yang, X. , Weng, X. , Xu, C. *et al* (2015) Coordinated regulation of vegetative and reproductive branching in rice. Proc. Natl. Acad. Sci. USA, 112, 15504–15509. 26631749 10.1073/pnas.1521949112 PMC4687603

[pbi12956-bib-0055] Werner, T. , Motyka, V. , Laucou, V. , Smets, R. , Van Onckelen, H. and Schmülling, T. (2003) Cytokinin‐deficient transgenic *Arabidopsis* plants show multiple developmental alterations indicating opposite functions of cytokinins in the regulation of shoot and root meristem activity. Plant Cell, 15, 2532–2550. 14555694 10.1105/tpc.014928 PMC280559

[pbi12956-bib-0056] Wood, C.C. , Robertson, M. , Tanner, G. , Peacock, W.J. , Dennis, E.S. and Helliwell, C.A. (2006) The *Arabidopsis thaliana* vernalization response requires a polycomb‐like protein complex that also includes VERNALIZATION INSENSITIVE 3. Proc. Natl. Acad. Sci. USA, 103, 14631–14636. 16983073 10.1073/pnas.0606385103 PMC1600011

[pbi12956-bib-0057] Wu, Y. , Wang, Y. , Mi, X.F. , Shan, J.X. , Li, X.M. , Xu, J.L. and Lin, H.X. (2016) The QTL *GNP1* encodes GA20ox1, which increases grain number and yield by increasing cytokinin activity in rice panicle meristems. PLoS Genet. 12, e1006386. 27764111 10.1371/journal.pgen.1006386 PMC5072697

[pbi12956-bib-0058] Yang, J. , Lee, S. , Hang, R. , Kim, S.R. , Lee, Y.S. , Cao, X. , Amasino, R. *et al* (2013) OsVIL2 functions with PRC2 to induce flowering by repressing *OsLFL1* in rice. Plant J. 73, 566–578. 23083333 10.1111/tpj.12057

[pbi12956-bib-0059] Yeh, S.Y. , Chen, H.W. , Ng, C.Y. , Lin, C.Y. , Tseng, T.H. , Li, W.H. and Ku, M.S. (2015) Down‐regulation of *Cytokinin oxidase 2* expression increases tiller number and improves rice yield. Rice, 8, 36. 10.1186/s12284-015-0070-5 PMC4671980 26643073

[pbi12956-bib-0060] Yoon, J. , Cho, L.H. , Kim, S.L. , Choi, H. , Koh, H.J. and An, G. (2014) The BEL1‐type homeobox gene SH5 induces seed shattering by enhancing abscission‐zone development and inhibiting lignin biosynthesis. Plant J. 79, 717–728. 24923192 10.1111/tpj.12581

[pbi12956-bib-0061] Yoon, J. , Cho, L.H. , Antt, H.W. , Koh, H.J. and An, G. (2017) KNOX Protein OSH15 Induces Grain Shattering by Repressing Lignin Biosynthesis Genes. Plant Physiol. 174, 312–325. 28351912 10.1104/pp.17.00298 PMC5411160

[pbi12956-bib-0062] Yoshida, N. , Yanai, Y. , Chen, L. , Kato, Y. , Hiratsuka, J. , Miwa, T. , Sung, Z.R. *et al* (2001) EMBRYONIC FLOWER2, a novel polycomb group protein homolog, mediates shoot development and flowering in Arabidopsis. Plant Cell, 13, 2471–2481. 11701882 10.1105/tpc.010227 PMC139465

[pbi12956-bib-0063] Zalewski, W. , Galuszka, P. , Gasparis, S. , Orczyk, W. and Nadolska‐Orczyk, A. (2010) Silencing of the *HvCKX1* gene decreases the cytokinin oxidase/dehydrogenase level in barley and leads to higher plant productivity. J. Exp. Bot. 61, 1839–1851. 20335409 10.1093/jxb/erq052

[pbi12956-bib-0064] Zhang, L. , Zhao, Y.L. , Gao, L.F. , Zhao, G.Y. , Zhou, R.H. , Zhou, R. , Zhang, B. *et al* (2012) *TaCKX6‐D1*, the ortholog of rice *OsCKX2*, is associated with grain weight in hexaploid wheat. New Phytol. 195, 574–584. 22670578 10.1111/j.1469-8137.2012.04194.x

[pbi12956-bib-0065] Zhang, Y.C. , Yu, Y. , Wang, C.Y. , Li, Z.Y. , Liu, Q. , Xu, J. , Liao, J.Y. *et al* (2013) Overexpression of microRNA OsmiR397 improves rice yield by increasing grain size and promoting panicle branching. Nat. Biotechnol. 31, 848–852. 23873084 10.1038/nbt.2646

