# Supplementary material for: Chromatin interacting factor OsVIL2 increases biomass and rice grain yield
Source: Plant Biotechnol J. 2018 Jun 26;17(1):178–87. doi: 10.1111/pbi.12956 (PMC6330541; doi:10.1111/pbi.12956)
Supplement: Supplementary file 1 — Figure S1 Confirmation of RNA sequencing by quantitative real‐time PCR. Figure S2 Transcript levels of LP, DST and DEP1 in WT and OsVIL2‐OX plants. [file PBI-17-178-s005.pptx]

## Slide 1
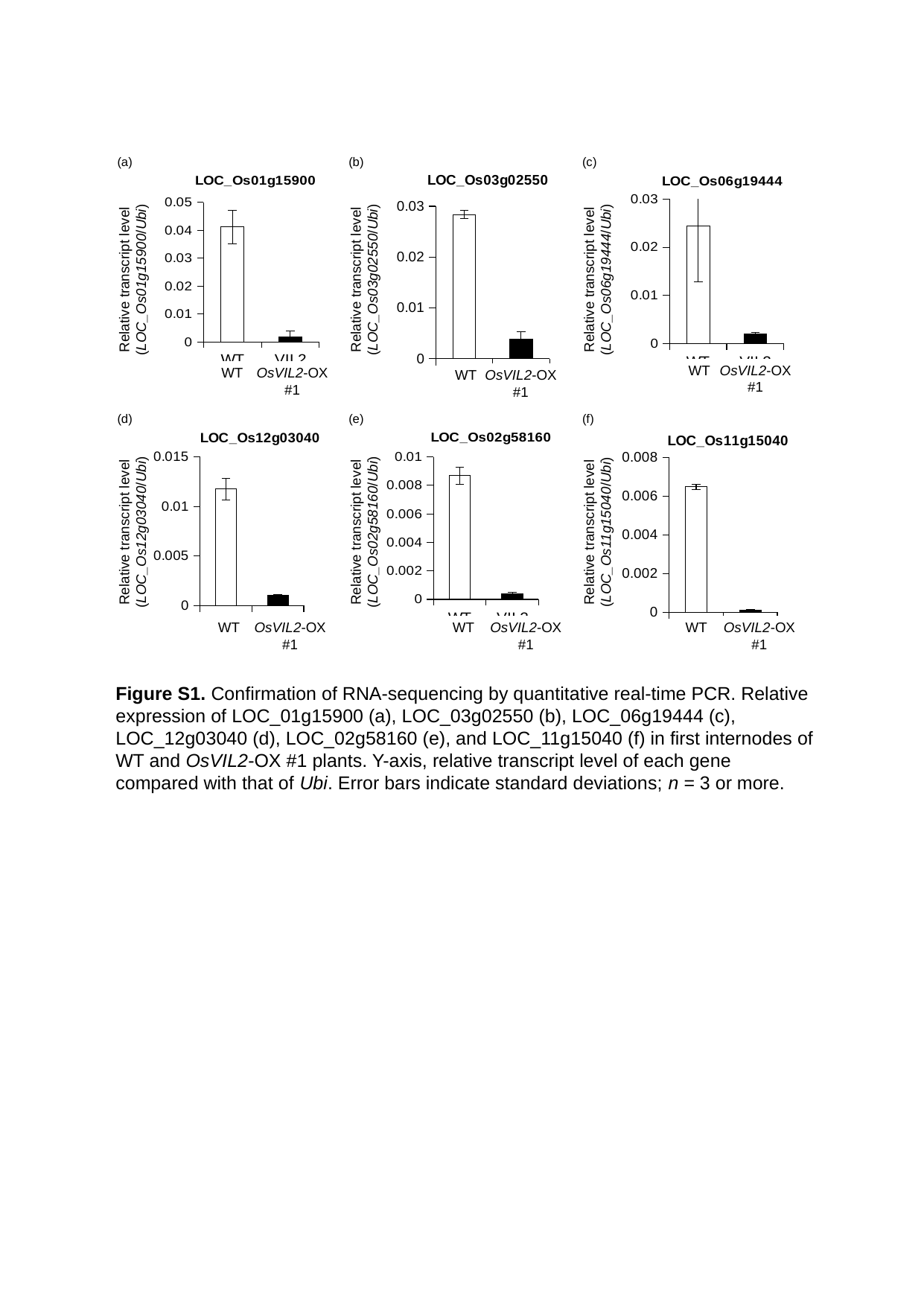

(a)
(b)
(c)
### Chart: LOC_Os01g15900
| Category | 01g15900 |
|---|---|
| WT | 0.04117133091796628 |
| VIL2 OX | 0.0016743754532440131 |WT
OsVIL2-OX #1
### Chart: LOC_Os06g19444
| Category | 06g19444 |
|---|---|
| WT | 0.02437653473065328 |
| VIL2 OX | 0.002053462725225471 |
### Chart: LOC_Os03g02550
| Category | 03g02550 |
|---|---|
| WT | 0.028366105360264737 |
| OsVIL2 OX | 0.003809673802286347 |Relative transcript level
(LOC_Os01g15900/Ubi)
Relative transcript level
(LOC_Os03g02550/Ubi)
Relative transcript level
(LOC_Os06g19444/Ubi)
WT
OsVIL2-OX #1
WT
OsVIL2-OX #1
(d)
(e)
(f)
### Chart: LOC_Os02g58160
| Category | 02g58160 |
|---|---|
| WT | 0.008678717293085303 |
| VIL2 OX | 0.00038529288327548647 |
### Chart: LOC_Os12g03040
| Category | 12g03040 |
|---|---|
| WT | 0.011744471214520467 |
| OsVIL2 OX | 0.001018644427031809 |
### Chart: LOC_Os11g15040
| Category | 11g15040 |
|---|---|
| WT | 0.00647968153832615 |
| VIL2 OX | 0.00011006839895096718 |Relative transcript level
(LOC_Os12g03040/Ubi)
Relative transcript level
(LOC_Os02g58160/Ubi)
Relative transcript level
(LOC_Os11g15040/Ubi)
WT
OsVIL2-OX #1
WT
OsVIL2-OX #1
WT
OsVIL2-OX #1
Figure S1. Confirmation of RNA-sequencing by quantitative real-time PCR. Relative expression of LOC_01g15900 (a), LOC_03g02550 (b), LOC_06g19444 (c), LOC_12g03040 (d), LOC_02g58160 (e), and LOC_11g15040 (f) in first internodes of WT and OsVIL2-OX #1 plants. Y-axis, relative transcript level of each gene compared with that of Ubi. Error bars indicate standard deviations; n = 3 or more.

## Slide 2
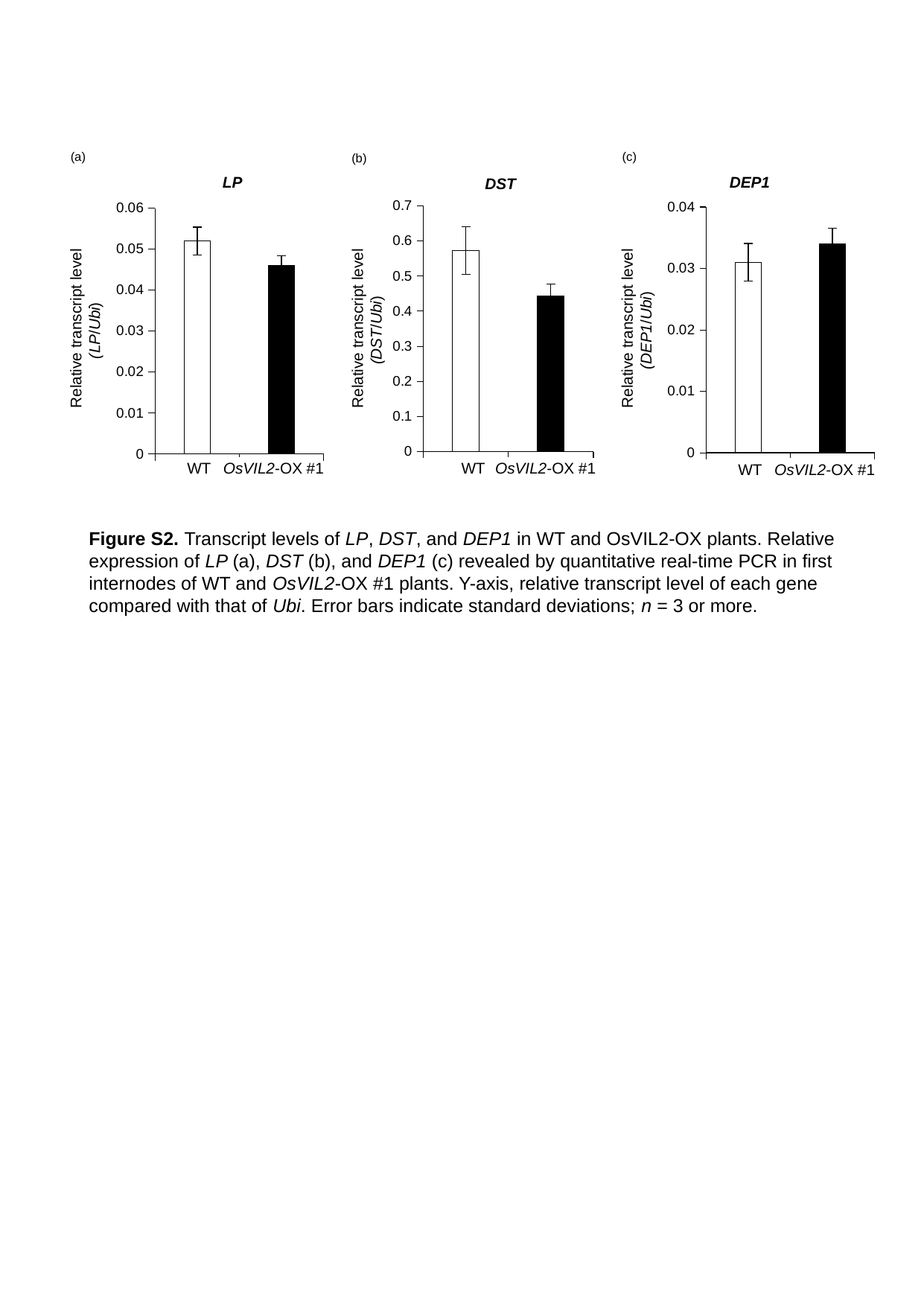

(a)
(c)
(b)
LP
DEP1
DST
### Chart
| Category | |
|---|---|
### Chart
| Category | |
|---|---|
### Chart
| Category | |
|---|---| Relative transcript level
(LP/Ubi)
 Relative transcript level
(DST/Ubi)
 Relative transcript level
(DEP1/Ubi)
WT
OsVIL2-OX #1
WT
OsVIL2-OX #1
WT
OsVIL2-OX #1
Figure S2. Transcript levels of LP, DST, and DEP1 in WT and OsVIL2-OX plants. Relative expression of LP (a), DST (b), and DEP1 (c) revealed by quantitative real-time PCR in first internodes of WT and OsVIL2-OX #1 plants. Y-axis, relative transcript level of each gene compared with that of Ubi. Error bars indicate standard deviations; n = 3 or more.
