# Supplementary material for: Chromatin interacting factor OsVIL2 increases biomass and rice grain yield
Source: Plant Biotechnol J. 2018 Jun 26;17(1):178–87. doi: 10.1111/pbi.12956 (PMC6330541; doi:10.1111/pbi.12956)
Supplement: Supplementary file 6 — Table S3 Classification of up‐ and down‐regulated genes in OsVIL2‐OX. The functional groups that are significantly abundant in the increased genes are indicated in blue. Table S6 Primers used in this study. [file PBI-17-178-s001.pptx]

## Slide 1
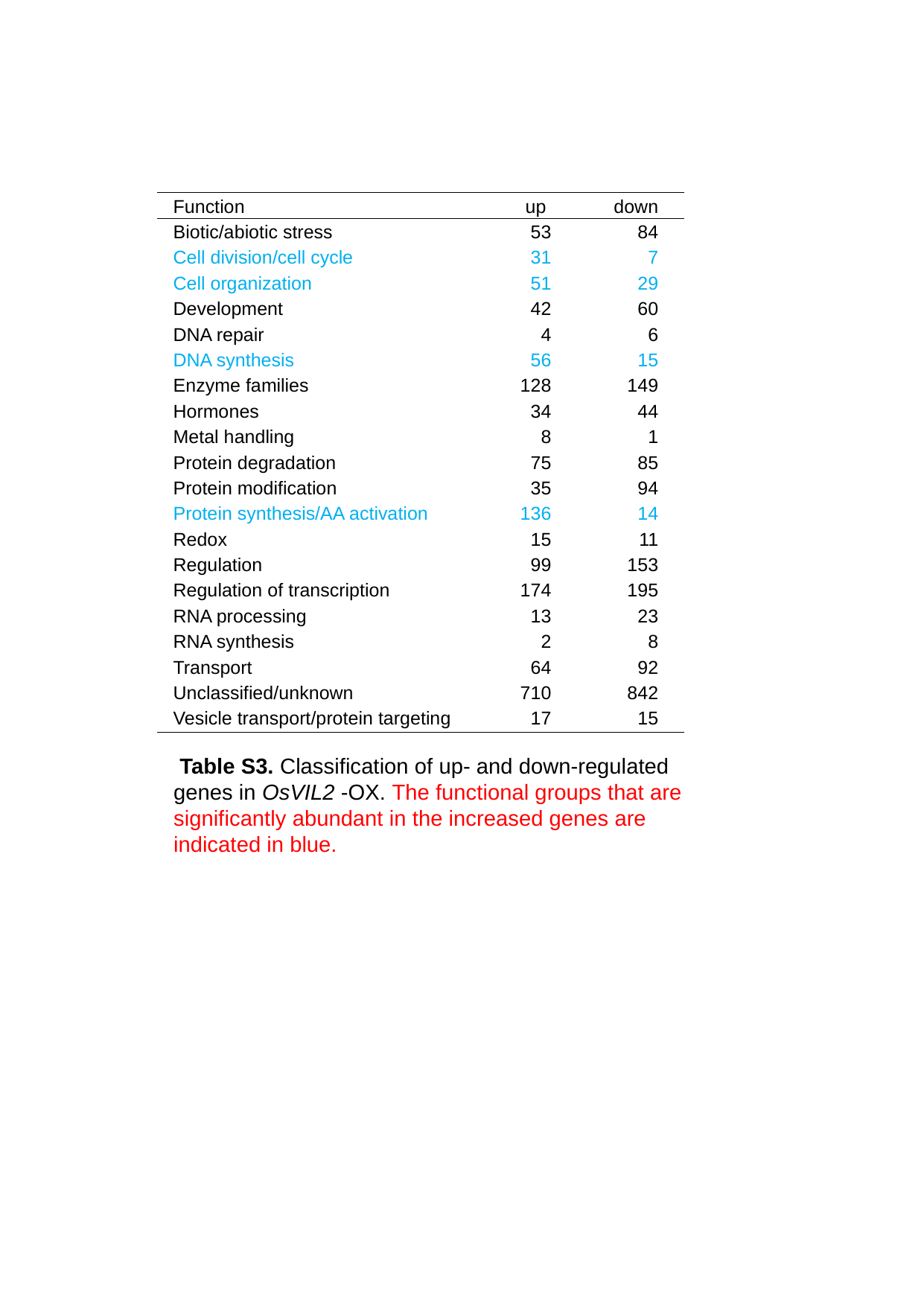

| Function | up | down |
| --- | --- | --- |
| Biotic/abiotic stress | 53 | 84 |
| Cell division/cell cycle | 31 | 7 |
| Cell organization | 51 | 29 |
| Development | 42 | 60 |
| DNA repair | 4 | 6 |
| DNA synthesis | 56 | 15 |
| Enzyme families | 128 | 149 |
| Hormones | 34 | 44 |
| Metal handling | 8 | 1 |
| Protein degradation | 75 | 85 |
| Protein modification | 35 | 94 |
| Protein synthesis/AA activation | 136 | 14 |
| Redox | 15 | 11 |
| Regulation | 99 | 153 |
| Regulation of transcription | 174 | 195 |
| RNA processing | 13 | 23 |
| RNA synthesis | 2 | 8 |
| Transport | 64 | 92 |
| Unclassified/unknown | 710 | 842 |
| Vesicle transport/protein targeting | 17 | 15 |
 Table S3. Classification of up- and down-regulated genes in OsVIL2 -OX. The functional groups that are significantly abundant in the increased genes are indicated in blue.

## Slide 2
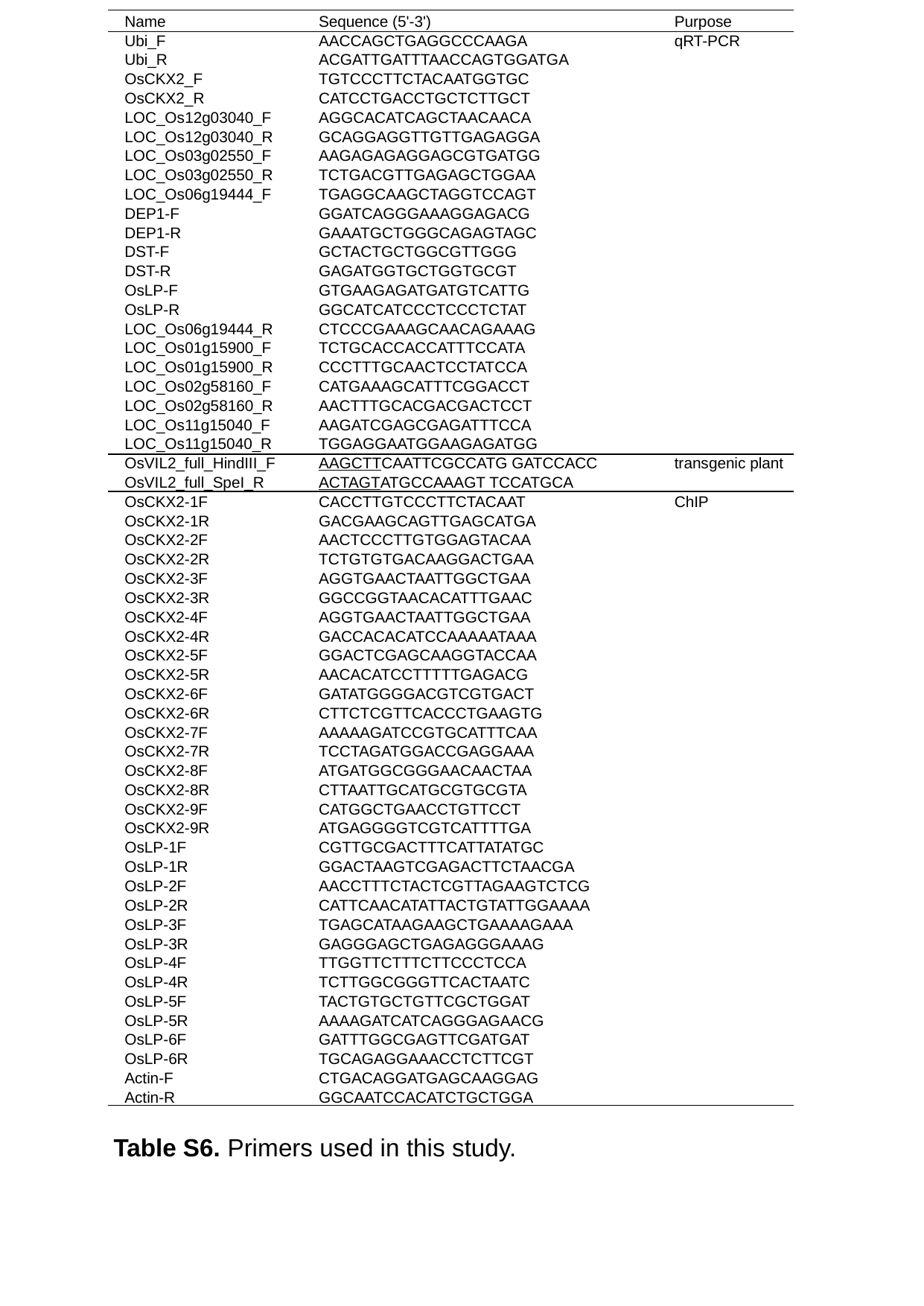

| Name | Sequence (5'-3') | Purpose |
| --- | --- | --- |
| Ubi\_F | AACCAGCTGAGGCCCAAGA | qRT-PCR |
| Ubi\_R | ACGATTGATTTAACCAGTGGATGA | |
| OsCKX2\_F | TGTCCCTTCTACAATGGTGC | |
| OsCKX2\_R | CATCCTGACCTGCTCTTGCT | |
| LOC\_Os12g03040\_F | AGGCACATCAGCTAACAACA | |
| LOC\_Os12g03040\_R | GCAGGAGGTTGTTGAGAGGA | |
| LOC\_Os03g02550\_F | AAGAGAGAGGAGCGTGATGG | |
| LOC\_Os03g02550\_R | TCTGACGTTGAGAGCTGGAA | |
| LOC\_Os06g19444\_F | TGAGGCAAGCTAGGTCCAGT | |
| DEP1-F | GGATCAGGGAAAGGAGACG | |
| DEP1-R | GAAATGCTGGGCAGAGTAGC | |
| DST-F | GCTACTGCTGGCGTTGGG | |
| DST-R | GAGATGGTGCTGGTGCGT | |
| OsLP-F | GTGAAGAGATGATGTCATTG | |
| OsLP-R | GGCATCATCCCTCCCTCTAT | |
| LOC\_Os06g19444\_R | CTCCCGAAAGCAACAGAAAG | |
| LOC\_Os01g15900\_F | TCTGCACCACCATTTCCATA | |
| LOC\_Os01g15900\_R | CCCTTTGCAACTCCTATCCA | |
| LOC\_Os02g58160\_F | CATGAAAGCATTTCGGACCT | |
| LOC\_Os02g58160\_R | AACTTTGCACGACGACTCCT | |
| LOC\_Os11g15040\_F | AAGATCGAGCGAGATTTCCA | |
| LOC\_Os11g15040\_R | TGGAGGAATGGAAGAGATGG | |
| OsVIL2\_full\_HindIII\_F | AAGCTTCAATTCGCCATG GATCCACC | transgenic plant |
| OsVIL2\_full\_SpeI\_R | ACTAGTATGCCAAAGT TCCATGCA | |
| OsCKX2-1F | CACCTTGTCCCTTCTACAAT | ChIP |
| OsCKX2-1R | GACGAAGCAGTTGAGCATGA | |
| OsCKX2-2F | AACTCCCTTGTGGAGTACAA | |
| OsCKX2-2R | TCTGTGTGACAAGGACTGAA | |
| OsCKX2-3F | AGGTGAACTAATTGGCTGAA | |
| OsCKX2-3R | GGCCGGTAACACATTTGAAC | |
| OsCKX2-4F | AGGTGAACTAATTGGCTGAA | |
| OsCKX2-4R | GACCACACATCCAAAAATAAA | |
| OsCKX2-5F | GGACTCGAGCAAGGTACCAA | |
| OsCKX2-5R | AACACATCCTTTTTGAGACG | |
| OsCKX2-6F | GATATGGGGACGTCGTGACT | |
| OsCKX2-6R | CTTCTCGTTCACCCTGAAGTG | |
| OsCKX2-7F | AAAAAGATCCGTGCATTTCAA | |
| OsCKX2-7R | TCCTAGATGGACCGAGGAAA | |
| OsCKX2-8F | ATGATGGCGGGAACAACTAA | |
| OsCKX2-8R | CTTAATTGCATGCGTGCGTA | |
| OsCKX2-9F | CATGGCTGAACCTGTTCCT | |
| OsCKX2-9R | ATGAGGGGTCGTCATTTTGA | |
| OsLP-1F | CGTTGCGACTTTCATTATATGC | |
| OsLP-1R | GGACTAAGTCGAGACTTCTAACGA | |
| OsLP-2F | AACCTTTCTACTCGTTAGAAGTCTCG | |
| OsLP-2R | CATTCAACATATTACTGTATTGGAAAA | |
| OsLP-3F | TGAGCATAAGAAGCTGAAAAGAAA | |
| OsLP-3R | GAGGGAGCTGAGAGGGAAAG | |
| OsLP-4F | TTGGTTCTTTCTTCCCTCCA | |
| OsLP-4R | TCTTGGCGGGTTCACTAATC | |
| OsLP-5F | TACTGTGCTGTTCGCTGGAT | |
| OsLP-5R | AAAAGATCATCAGGGAGAACG | |
| OsLP-6F | GATTTGGCGAGTTCGATGAT | |
| OsLP-6R | TGCAGAGGAAACCTCTTCGT | |
| Actin-F | CTGACAGGATGAGCAAGGAG | |
| Actin-R | GGCAATCCACATCTGCTGGA | |
 Table S6. Primers used in this study.
